# Effect of Bariatric Surgery on Survival and Hospitalizations in Patients with Severe Obesity. A Retrospective Cohort Study

**DOI:** 10.3390/nu13093150

**Published:** 2021-09-09

**Authors:** Enrica Migliore, Amelia Brunani, Giovannino Ciccone, Eva Pagano, Simone Arolfo, Tiziana Rosso, Marianna Pellegrini, Paolo Capodaglio, Mario Morino, Ezio Ghigo, Simona Bo

**Affiliations:** 1 Unit of Clinical Epidemiology, CPO, “Città della Salute e della Scienza” Hospital of Torino, 10126 Torino, Italy; enrica.migliore@cpo.it (E.M.); gianni.ciccone@cpo.it (G.C.); eva.pagano@cpo.it (E.P.); tiziana.rosso@cpo.it (T.R.); 2 Rehabilitation Medicine Unit, IRCCS Istituto Auxologico Italiano Piancavallo (Verbania), 28921 Oggebbio, Italy; brunani@auxologico.it (A.B.); paolo.capodaglio@unito.it (P.C.); 3 Department of Surgical Sciences, University of Torino, 10126 Torino, Italy; simone.arolfo@unito.it (S.A.); mario.morino@unito.it (M.M.); 4 Department of Medical Sciences, University of Torino, 10126 Torino, Italy; marianna.pellegrini@unito.it (M.P.); ezio.ghigo@unito.it (E.G.)

**Keywords:** bariatric surgery, hospitalization, overall survival, Roux-en-Y gastric bypass, sleeve gastrectomy

## Abstract

Bariatric surgery (BS) confers a survival benefit in specific subsets of patients with severe obesity; otherwise, effects on hospital admissions are still uncertain. We assessed the long-term effect on mortality and on hospitalization of BS in patients with severe obesity. This was a retrospective cohort study, including all patients residing in Piedmont (age 18–60 years, BMI ≥ 40 kg/m^2^) admitted during 2002–2018 to the Istituto Auxologico Italiano. Adjusted hazard ratios (HR) for BS were estimated for mortality and hospitalization, considering surgery as a time-varying variable. Out of 2285 patients, 331 (14.5%) underwent BS; 64.4% received sleeve gastrectomy (SG), 18.7% Roux-en-Y gastric bypass (RYGB), and 16.9% adjustable gastric banding (AGB). After 10-year follow-up, 10 (3%) and 233 (12%) patients from BS and non-BS groups died, respectively (HR = 0.52; 95% CI 0.27–0.98, by a multivariable Cox proportional-hazards regression model). In patients undergoing SG or RYGB, the hospitalization probability decreased significantly in the after-BS group (HR = 0.77; 0.68–0.88 and HR = 0.78; 0.63–0.98, respectively) compared to non-BS group. When comparing hospitalization risk in the BS group only, a marked reduction after surgery was found for all BS types. In conclusion, BS significantly reduced the risk of all-cause mortality and hospitalization after 10-year follow-up.

## 1. Introduction

Obesity is a recognized prevalent disease associated with an increased risk for type 2 diabetes mellitus, cardiovascular, kidney, lung, and osteoarticular diseases, many cancers, and a reduced lifespan [1]. Owing to the frequent unsatisfactory outcomes of the conservative treatments (i.e., dietary/behavioral, and pharmacological interventions), bariatric surgery (BS) remains a safe and effective intervention for higher risk patients with obesity [2]. The reduction in the risk of all-cause mortality by BS varied from 30 to 90% [2], with a 38% risk reduction being reported by a large meta-analysis of non-randomized comparative studies based upon registry data [3]. An adjusted median life expectancy of 3.0 years longer was reported in patients treated with BS in comparison to controls [4]. Nevertheless, in patients with obesity, the rate of death for causes other than diseases, such as accidents or suicides, resulted in being higher after BS [5,6], and the relative risk of death remained higher than in the general background population, even after BS [4,6]. Other authors reported no beneficial effects on long-term mortality in patients with a younger age and lower BMI [7,8], or extremely high BMI [9]. Recently, a large meta-analysis described a much more pronounced survival benefit in patients with diabetes mellitus, with a median 9-year longer life expectancy after BS than the non-surgical group, while patients without diabetes showed a 5-year life expectancy gain [10].

More uncertain are the consequences of BS on hospital admissions and healthcare resource use. Many studies reported lower hospital admissions after BS [11,12,13,14,15], suggesting beneficial effects on the chronic conditions characterizing individuals with obesity, such as type 2 diabetes mellitus, cardiovascular risk factors and diseases, chronic sub-clinic inflammation, and some types of cancer [16,17,18,19,20,21,22,23]. Other authors reported an increased request for inpatient and nonprimary outpatient care during the first 6-year period, but not thereafter in post-BS patients when compared to controls [24]. Furthermore, an overall increase in the number of visits and direct medical costs [25], a higher risk of hospital admission for late adverse events of BS (above all, after gastric bypass) [26] and an overall increased risk for hospitalization have been described [27,28,29,30]. Many of these studies referred to cohort submitted to BS in the nineties, with outdated procedures and a lesser use of laparoscopic techniques [24,30] with an increased risk of hospitalization due to surgery complications [13,28]. Finally, the fact that most patients undergoing BS received a weight-loss program and monitoring, whereas a similar intervention was not always performed in control cohorts was suggested as a potential source of bias of the available literature, potentially leading to an overestimation of BS benefits [3]. We therefore have studied a large cohort of patients with severe obesity initially treated within a specialized institute, including a standardized lifestyle approach, to compare the long-term outcomes of those undergoing BS (BS group) towards individuals not undergoing BS (non-BS group) during a 10-year follow-up period. The primary outcome was between-group difference in overall survival. Secondary outcomes were determinants of BS surgery, between-group differences in the hospitalization rates and within-group variations in length, and complexity of hospital admissions before and after BS.

## 2. Materials and Methods

This retrospective cohort study used clinical data collected at baseline and administrative databases for outcome assessment. All the patients residing in Piedmont (North-Western Italy) admitted from 2002 to 2018 for severe obesity or its complications to the Istituto Auxologico Italiano (IAI) in Piancavallo, a specialized center for the treatment of severe obesity, were included. The Institute is a tertiary care center, accredited by the European Association for the Study of Obesity (EASO) as a Centre for Obesity Management. Patients are admitted for clinical investigation of obesity with BMI ≥ 40 kg/m^2^ (or 35–40 kg/m^2^ in presence of comorbidities), and without known comorbidities or conditions reducing the life expectancy. A multidisciplinary program (nutritional, physical and psychological intervention) is applied for about 1 month; then patients are followed-up for 12–16 months, based on the individual needs.

For this analysis, the inclusion criteria were restricted to patients with age 18–60 years and BMI ≥ 40 kg/m^2^ to select a population eligible for BS.

### 2.1. Objectives

The primary objective of the study was the association between BS and all-cause mortality. Secondary objectives were to assess the hospitalization rates of patients undergoing BS versus the non-BS group, and to compare hospitalization rates, length of stay, and complexity of hospital admissions before and after surgery in the BS group (henceforth defined as pre-BS and post-BS hospitalizations).

### 2.2. Data Collection and Measurements

Patient data were collected from the medical records of the IAI archive. In particular, the following data were abstracted: demographic information, smoking habits, values of weight, height, waist circumference, arterial blood pressure, fasting blood concentrations of glucose, total cholesterol, HDL cholesterol, triglycerides, uric acid, and creatinine. In the case of multiple admissions to the IAI, data relative to the first admission were considered.

At the IAI, anthropometric measures were standardized, in particular, body-weight (kg) and body height (cm) were measured to the nearest 0.1 kg and 0.5 cm respectively, using a mechanical column scale (Scale-Tronix, Wheaton, IL, USA) and a stadiometer (Scale-Tronix, Wheaton, IL, USA). Waist circumference (cm) was measured with a non-elastic tape at the level of the umbilicus. Systolic and diastolic blood pressures were recorded in resting condition, using an oscillometric sphygmomanometer. Blood samples for glucose, total cholesterol, HDL cholesterol, triglycerides, uric acid, and creatinine were drawn in the morning after an overnight fast and evaluated by routine standard hospital methods and procedures (Modular-Roche) in the institute laboratory. Diabetes mellitus was defined in the presence either of hypoglycemic treatment or fasting glucose level ≥ 126 mg/dL.

For each patient, all subsequent hospital discharge records (HDRs) from any hospital in Italy were identified from the regional database by a deterministic record-linkage procedure through the unique anonymous identifier code. Hospitalizations were classified according to Major Diagnostic Categories (MDC) of the Medicare Diagnosis-Related Groups (DRG) classification (v. 24.0).

The coded version of the Charlson Comorbidity Index, based on the International Classification of Diseases, ninth revision, Clinical Modification (ICD9-CM) codes was calculated for each patient from the HDR of the index admission [31].

BS was identified according to presence of a main diagnosis of morbid obesity (ICD-9-CM 278.01) and one of the following procedures: 43.89, 44.31, 44.38, 44.95, and 44.99. In the case of generic codes, the full HDR was checked to confirm the correct assignment. Type of surgery was classified as: Roux-en-Y gastric bypass (RYGB), (ICD9-CM codes 44.31, 44.38) sleeve gastrectomy (SG), (ICD9-CM codes 43.89, 44.99), and adjustable gastric banding (AGB), (ICD9-CM code 44.95). All were laparoscopic procedures. Guideline recommendations, characteristics and willingness of the patients, the local surgeon expertise and local practice determined the procedure choice. To ensure a minimum follow-up period (at least 2 years), only BS procedures performed within the year 2018 were considered for the analyses. Information on vital status until August 2020 was collected through a record linkage with the registry of the residents in the Piedmont Region by means of the unique anonymous code.

### 2.3. Ethical Aspects

All individual data were recorded anonymously in a computerized database and processed according to the principles of minimization, confidentiality and security provided for by the legislation (EU Regulation 2016/679 and subsequent amendments) and only for the purposes of the protocol. Data anonymization was guaranteed by working with encrypted personal identification codes. For organizational reasons and the high risk of introducing a selection bias in the study, obtaining the consent to the processing of data by all the members of the cohort was not feasible. The study protocol was approved by the Ethics Committee of the IAI (approval code #2019_10_22_01).

### 2.4. Statistical Analyses

A minimum sample size of 1100 patients was required to ensure a statistical power of around 85% to estimate a 40% relative reduction in mortality (similar to that reported in the literature [32] during a follow-up lasting about 10 years and assuming that about 5% of patients underwent BS during the study).

Continuous or categorical variable distributions between groups were compared through the Wilcoxon rank-sum test or the Chi square test, respectively.

To identify factors associated with BS, we modelled the cumulative BS incidence, treating the mortality as a competing risk event. We censored the observation of patients when they reach 65 years, the age beyond which the probability of intervention is around 0. Cumulative incidence of BS was assessed using a multivariable competing risk regression model for the sub-distribution hazards (SHRs) and 95% CI fitted to baseline data according to the method of Fine and Gray [33]. Overall survival was calculated from date of discharge from the IAI to death for any cause or to the end of follow-up. A multivariable Cox proportional-hazards regression model was used to estimate the association, reported as hazard ratios (HR) and 95% confidence intervals (95% CI), between prognostic factors or variables associated with BS and survival. BS was included in the model as a time-dependent variable to avoid a survival time bias. For variables with missing values >5% (smoking, alcohol, waist circumference), a separate category (“not defined”) was created and included in the multivariable models. The role of BS was estimated overall and by type of surgery.

Hospitalization rates, excluding the surgical admission for BS, were estimated as number of admissions, both overall and by type of care (medical or surgical), over the person/years at risk of each patient. Hospitalizations were described in relation to median length of stay and mean DRG weight. Total DRG tariffs per person/years were calculated in euros.

To take into account the possibility of repeated hospitalizations, HRs, and 95% CI were estimated through an extension of the Cox regression model (Prentice, Williams and Peterson Total Time, PWP-TT [34], with stratification on the number of accumulated previous admissions. The risk of hospitalization was estimated between groups (non-BS vs. BS group) by adjusting for the available set of covariates, and within groups (hospital admissions pre and post BS), separately by type of surgery performed (AGB, SG, and RYGB). Statistical analyses were performed with Stata 15 (StataCorp LP, College Station, TX, USA).

This study was reported according to the Strengthening the Reporting of Observational Studies in Epidemiology, (STROBE) reporting guideline for cohort studies.

## 3. Results

Between January 2002 and December 2018, a total of 2285 patients with severe obesity were enrolled in the IAI cohort. Out of them, 331 (14.5%) underwent BS after a median of 4.6 years; 213 (64.4%) received SG, 62 (18.7%) RYGB, and 56 (16.9%) AGB. Table 1 shows the socio-demographic and clinical characteristics of the study cohort at baseline. Patients undergoing BS during the study period were younger, with a higher education level, and a lower systolic blood pressure, fasting glucose values, diabetes mellitus prevalence and comorbidity index than those without BS. Patients undergoing AGB were mainly females with a lower weight than those undergoing SG or RYGB. No other significant difference in the baseline characteristics was evident among types of BS.

By taking into account the competitive risk of death, the cumulative incidence of BS was 16.6% (95% CI 14.9–18.5) at the median follow-up time (10.6 years) (Figure S1). At a multivariable competing risk regression model, older patients had a lower probability to undergo BS (SHR = 0.97 per year, 95% CI 0.96–0.98) and BMI was slightly associated with a higher probability of BS (SHR = 1.01 per unit, 95% CI 1.00–1.02) (Table S1). The category “not defined” smoking was associated with a lower probability of BS, suggesting a non-random distribution of missing data for this exposure.

### 3.1. BS and All-Cause Mortality

The mean follow-up time of the whole sample was 10.2 years (SD = 4.8), with 23,221 person/years of observation (6964 for males and 16,257 for females). During follow-up, 243 patients (11%) died, 10 (3%) after BS and 233 (12%) in the non-BS group. We observed an overall survival at 5 and 10 years of 95.9% (95% CI 95.0–96.7) and 91.1% (95% CI 89.6–92.3), respectively (Figure S2A). In Figure S2B, overall survival was plotted for patients after BS and for the same patients before surgery and those never operated on (*p* = 0.009, log-rank test). Table 2 shows the results of the analyzed variables on survival. The risk of death was lower in the BS groups in both univariate (HR = 0.36; 95% CI 0.19–0.67) and multivariable analyses (HR = 0.52; 0.27–0.98). Age, smoking, BMI, multiple comorbidities, and low cholesterol values were negative prognostic factors, while female gender was associated with a lower probability of all-cause mortality. When considering the type of surgical intervention, SG only was associated with a reduced mortality risk (HR = 0.39; 95% CI 0.17–0.89), while no association was observed for RYGB (HR = 0.97; 0.35–2.66). The absence of association was probably due to the small sample size in the RYGB group. The effect on mortality for AGB was not estimable because no events occurred in this group.

Results were confirmed after adjusting for the presence of diabetes instead of glucose values.

### 3.2. BS and Hospital Admission

During the observation period 10,762 hospital admissions were identified, with a median number of 3 hospitalizations per patient (IQR: 2–6). Excluding 331 admissions for BS, 83.5% (*n* = 8707) of the hospitalizations occurred in the non-BS group, whereas in the subgroup of patients undergoing BS 1013 (10.6%) pre-surgery hospitalizations and 711 (6.8%) post-surgery hospitalizations were recorded.

During the follow-up period, the main causes of hospitalization were due to endocrine, nutritional and metabolic diseases/disorders (30.2% and 40.8% in non-BS and BS groups, respectively). The distribution of causes was similar among groups until BS. In the post-BS period, regardless of the type of surgery performed, the frequency of hospitalizations for nutritional problems decreased, while in-hospital admissions for musculoskeletal system disorders, mental disorders, and digestive tract disorders increased. A total of 329 hospitalizations were related to cancer, almost entirely recorded in the non-BS group (94%); main diagnoses were neoplasms of the reproductive system (22.2%), myeloproliferative diseases (17.1%), and digestive cancers (10%) (data not shown).

The characteristics of hospitalizations by BS were reported in Table 3. Patients from the BS group showed higher risks of hospitalization (0.61 per person/years) compared to the non-BS group in the pre-surgery period (0.46 per person/years), but a close rate in the post-surgery period (0.49 per person/years), with a relevant decrease in medical admissions and an increase in surgical ones. Median length of stay was reduced post-BS (from 14 to 6 days), with a more complex level of care (mean DRG weight: 0.76 before-BS and 1.13 after-BS). Total DRG tariffs per person/years decreased from 2508 euros before-BS to 1891 euros after BS.

The risk of hospitalization in the non-BS group and in the BS-group, both before/after surgery and by type of surgery, was reported in Table 4. The risk of pre-BS hospitalization was higher than in the non-BS group (HR = 1.16; 95% CI 1.08–1.24), in both SG and RYGB surgery, but not in the AGB group. In those undergoing SG or RYGB, the probability of hospitalization decreased significantly after surgery (HR = 0.77; 95% CI 0.68–0.88 and HR = 0.78; 0.63–0.98, respectively), whereas for AGB the reduction was not statistically significant (HR = 0.92; 0.72–1.18) when compared with the non-BS group. However, when comparing risk of hospitalization before and after BS only for patients undergoing BS (within-group analysis) including admissions for reinterventions and plastic surgery, a lower probability was found for all types of surgery in the post-BS period (HR = 0.57; 95% CI 0.46–0.71 for SG, HR = 0.43; 0.43–0.72 for AGB, and HR = 0.51; 0.34–0.77 for RYGB) when compared to the pre-BS period.

## 4. Discussion

In this 10-year follow-up cohort study, BS was associated with approximately half the risk of all-cause mortality and a more than 20% reduction in the risk of hospitalization in patients with severe obesity compared to individuals with the same degree of obesity who had received the same standardized lifestyle approach for at least 1 year, but had not undergone BS.

### 4.1. Overall Survival

Several observational studies and a few randomized controlled trials, and meta-analyses have reported a survival benefit for BS. In particular, a very recent, large, and robust meta-analysis of individual data from matched cohort and case-control studies (with 174,772 participants) reported an overall HR = 0.51, which was remarkably similar to our overall estimate [10]. Intriguingly, we found a very low number of deaths in the BS group in the long-term when compared to non-BS controls, in line with another large Italian cohort study [35]. The reduction of mortality could be justified by the substantial weight loss, leading to the improvement/remission of dysmetabolic abnormalities, cardiovascular, respiratory, renal outcomes, cancer reduced risk, and better motility and quality of life reported after BS [3,12,16,17,18,20,22,23,36,37]. This is not surprising if the effects of excess fat deposition, such as increased oxidative stress, chronic inflammation, endothelial dysfunction, ectopic lipid deposition, impairments in metabolism and heart, lung, and kidney functions, are considered together with the potential effects of BS in reversing/reducing most of these unfavorable conditions [2].

### 4.2. Overall Hospitalizations

Data relative to hospital admissions and healthcare resource use after BS are highly controversial. Despite the undeniable health benefits, there are several studies reporting an increased use of health resources and costs after BS [25,38,39]. Indeed, several studies were assessed in a short period of time [25,27,28,38,39], while in the medium to long-term, a trend in the reduction of health resource and hospital use was uniformly reported, with figures that were at least similar, or even lower [14,24,25,39,40,41,42]. Accordingly, we found a significant reduction in the hospitalization rate in the long-term after BS. Intriguingly, this reduction occurred even if patients from the BS group showed an increased risk for hospitalization before surgery compared to the non-BS group. We have specifically assessed the before–after hospitalization rates and characteristics within the BS group, thus allowing the patients to be their own controls and overcoming the issue of measured and unmeasured confounders. In this case, the increase in age of the cohort should have rather increased the risk of hospitalization while we observed a clear reduction. In particular, after BS, we found a relevant decrease in the medical admissions, such as hospitalizations for nutritional problems and cancer, while surgical admissions increased. These results are in line with the reported differences in admissions in the preoperative and postoperative periods, with diagnoses much more likely to be obesity-related before BS and procedure-related complications after BS [30].

We found shorter in-hospital stays after BS, while other long-term studies reported increased mean cumulative hospital days in surgery patients, which progressively declined over time until they became similar [24]. However, older studies included other surgical procedures and/or open techniques, which are more invasive and associated with a higher proportion of surgery-related complications and hospital use [13,24,28]. Indeed, recent cohort studies found a shorter length of stay and overall better hospitalization outcomes after BS [43,44]. In-hospital admissions of our BS cohort, although less frequent and shorter in duration, were more complex, as documented by the increase in the DRG’s weight. This may be due to the increase in surgical admissions, mainly for reinterventions, or for plastic surgery. However, the average DRG tariffs per person/years decreased after surgery, thus confirming the overall benefits of BS in terms of use of hospital resources.

### 4.3. Differences among AGB, SG, and RYGB

We found a significant difference in overall survival among the types of operation. Patients undergoing RYGB showed the same mortality risk of non-BS individuals, while AGB and SG were responsible of the significant increase in overall survival. However, the small number of events in the BS group (10 deaths only) did not allow us to have sufficient data to discuss the differences by type of surgery.

All three procedures resulted in a reduced before–after risk of hospitalization, but only SG and RYGB showed a lower risk with respect to the non-BS group. This is noteworthy since both of these two groups displayed an increased risk of in-hospital admissions before surgery than the non-BS group. The increased risk of reoperation after AGB is well known, mostly due to pouch dilatation with band slippage, gastric erosion, and food intolerance [45]. This, together with the subsequent poor weight loss and the high rate of subsequent revisional surgery [46], has made this surgical technique less employed at present.

Higher metabolic and cardiovascular benefits after RYGB than after SG were described [22,23,37,47,48], probably due to the higher weight loss as well as the increased secretion of glucagon-like peptide (GLP)-1 or altered gut–brain signaling in the former [23,37]. However, the magnitude of the weight loss is not currently recognized as the main determinant of the beneficial effects of BS on obesity and its complications, since many weight-independent benefits of gastrointestinal operations have been suggested, such as the restoration of obesity-induced somatotropic axis alterations, the improvement in pancreatic islet function and the modulation of gastrointestinal hormones, bile secretion, circulating adipokines, and gonadal axis, together with the favorable changes in the gut microbiota composition [49,50].

On the other hand, a higher incidence of complications after RYGB have been reported, such as fall-related accidents, kidney stones and diseases, gastrointestinal disorders, micronutrient deficiencies, endocrine derangements [26,51,52] with respect to SG, which is considered to be a less technically demanding procedure. Furthermore, an increase in costs and hospitalization rates [26,29,30,38,39,41,53] were described after RYGB, with increased percentages of readmission, re-operation, and subsequent invasive procedures (frequently, complications due to the procedures, such as ventral hernia repair, small bowel obstruction, and gastric revision). A recent meta-analysis of randomized controlled trials, however, failed in drawing any conclusions regarding the long-term comparative effectiveness between RYGB and SG beyond 3 years [54]. Accordingly, we found a similar decrease in both in-hospital admissions than non-BS groups and before–after hospitalizations with the two procedures.

### 4.4. Clinical Implications

Metabolic-bariatric surgery is an approved treatment for severe and complicated obesity; however there is still only a low percentage of patients who are potentially candidates of BS that are treated. Guidelines suggest selecting the bariatric procedure by basing on “individualized goals of therapy (e.g., weight-loss target and/or improvements in specific obesity-related complications), available local-regional expertise (obesity specialists, bariatric surgeon, and institution), patient preferences, personalized risk stratification that prioritizes safety, and other nuances as they become apparent” [55]. The long-term mortality and hospitalization use after each procedure might be further aspects worthy of consideration when making a choice about the surgical procedure.

### 4.5. Limitations and Strengths

The observational design of the present study does not allow strong inferences to be drawn, residual or unmeasured confounders could have influenced the findings of this retrospective study, even if we had assessed patients from a homogeneous cohort in order to reduce extra variability in the cohort. Still, some individuals might have been ineligible for BS and imbalances which existed at baseline between BS and non-BS groups, but great care was taken in adjusting for all the baseline characteristics. The extremely low number of deaths in the post-BS patients and the small sample size of the RYGB group might have been insufficient to find significant differences in this group. Concerns might arise about the generalizability of these results owing to the enrolment from a single specialized center; however, after the first year from enrolment, patients were treated and operated on in a wide range of structures reflecting a “real world” scenario, that may be generalizable to routine clinical practice. Finally, the causes of mortality could not be determined.

Strengths of the study were a centralized anthropometric assessment measured at a single center by trained professionals, and the length and completeness of the follow-up. The study was based on a large cohort, with baseline information available on several well-recognized risk factors for subsequent morbidity and mortality. The statistical analyses were conducted trying to reduce the immortal time bias and the effects of several potential confounders. Finally, different to previous studies, where the control cohorts of non-BS patients with obesity were derived from the general population and likely did not receive the same monitoring and treatment of the patients submitted to BS [3,4,7,8,18], our cohort of patients received the same 1-month standardized multidisciplinary program and a follow-up for 12–16 months. This did not prevent the two groups (BS and non-BS) from receiving subsequent different lifestyle and pharmacological treatment, which could have impacted on the measured outcomes. However, it guaranteed that at least all participants received the same lifestyle education over a medium-term period.

## 5. Conclusions

BS was associated with a significant reduction in the risk of all-cause mortality and hospitalization after 10-year follow-up. Although the risks and possible adverse effects of surgery should be carefully considered, BS is a treatment option in patients with severe obesity with relevant long-term benefits.

## Figures and Tables

**Table 1:** Socio-demographic and clinical characteristics of the study cohort at baseline and of patients undergoing bariatric surgery (BS) intervention during follow-up, by type of BS.

		Bariatric Surgery during Follow-Up			Types of BS			
	Total	No	Yes		AGB	SG	RYGB	
	(*n* = 2285)	(*n* = 1954)	(*n* = 331)	*p*	(*n* = 56)	(*n* = 213)	(*n* = 62)	*p*
Age at cohort enrolment, years (%)								
18–24	143 (6.3)	102 (5.2)	41 (12.4)	<0.001	7 (12.5)	23 (10.8)	11 (17.7)	0.578
25–34	285 (12.5)	228 (11.7)	57 (17.2)		9 (16.1)	36 (16.9)	12 (19.4)	
35–44	483 (21.1)	391 (20.0)	92 (27.8)		17 (30.4)	55 (25.8)	20 (32.3)	
45–54	770 (33.7)	664 (34.0)	106 (32.0)		19 (33.9)	73 (34.3)	14 (22.6)	
>55	604 (26.4)	569 (29.1)	35 (10.6)		4 (7.1)	26 (12.2)	5 (8.1)	
Age, mean (SD)	45.6 (11.2)	46.4 (11.0)	40.7 (11.3)	<0.001	40.7 (11.2)	41.3 (11.4)	38.5 (11.2)	0.227
Females (%)	1528 (66.9)	1296 (66.3)	232 (70.1)	0.178	48 (85.7)	137 (64.3)	47 (75.8)	0.004
Educational level (%)								
Degree	86 (3.8)	74 (3.8)	12 (3.6)	<0.001	0 (0.0)	9 (4.3)	3 (4.8)	0.534
High school	718 (31.7)	612 (31.6)	106 (32.2)		19 (33.9)	64 (30.3)	23 (37.1)	
Intermediate school	1099 (48.5)	916 (47.2)	183 (55.6)		33 (58.9)	121 (57.3)	29 (46.8)	
Elementary school or less	365 (16.1)	337 (17.4)	28 (8.5)		4 (7.1)	17 (8.1)	7 (11.3)	
Smoking (%)	597 (27.6)	514 (28.0)	83 (25.5)	0.367	15 (27.3)	54 (25.8)	14 (23.0)	0.856
Alcohol (%)	798 (37.4)	692 (38.1)	106 (33.0)	0.081	13 (23.6)	78 (38.0)	15 (24.6)	0.039
Charlson Index (%)								
0	1409 (61.7)	1171 (59.9)	238 (71.9))	<0.001	40 (71.4)	154 (72.3)	44 (71.0)	0.499
1	659 (28.8)	590 (30.2)	69 (20.8)		10 (17.9)	43 (20.2)	16 (25.8)	
≥2	217 (9.5)	193 (9.9)	24 (7.3)		6 (10.7)	16 (7.5)	2 (3.2)	
Weight (kg), mean (SD)	126.0 (33.7)	125.8 (35.1)	127.7 (23.8)	0.345	119.2 (15.3)	129.3 (24.6)	129.7 (25.9)	0.013
Height (cm), mean (SD)	164.1 (37.6)	164.3 (40.5)	163.5 (9.0)	0.717	161.3 (7.3)	164.1 (9.5)	163.3 (8.8)	0.127
BMI (kg/m^2^), mean (SD)	46.9 (7.3)	46.9 (7.4)	47.4 (6.6)	0.234	45.8 (5.9)	47.6 (6.6)	48.3 (7.2)	0.104
Waist (cm), mean (SD)	129.2 (15.7)	129.3 (15.3)	128.7 (17.6)	0.489	124.1 (14.6)	130.6 (15.5)	126.6 (24.5)	0.031
Fasting glucose (mg/dL), mean (SD)	107.8 (44.6)	109.2 (46.6)	99.2 (28.7)	<0.001	98.4 (24.5)	99.8 (31.1)	98.0 (23.5)	0.883
Creatinine (mg/dL), mean (SD)	0.8 (1.3)	0.8 (1.4)	0.8 (0.7)	0.792	0.7 (0.2)	0.8 (0.8)	0.8 (0.6)	0.675
Uric acid, mean (SD)	5.6 (3.6)	6.6 (3.8)	6.4 (1.5)	0.434	6.2 (1.2)	6.4 (1.5)	6.4 (1.6)	0.557
Total cholesterol (mg/dL), mean(SD)	197.2 (88.5)	197.9 (94.3)	193.3 (39.0)	0.385	192.8 (38.3)	192.7 (40.6)	195.8 (34.0)	0.853
HDL cholesterol (mg/dL), mean (SD)	45.4 (12.8)	45.3 (12.7)	45.9 (13.0)	0.387	47.3 (14.7)	45.0 (12.9)	47.9 (11.6)	0.217
Triglycerides (mg/dL), mean (SD)	154.5 (97.3)	155.4 (99.9)	149.1 (80.7)	0.279	153.6 (79.4)	148.0 (82.7)	149.2 (75.8)	0.899
Systolic BP (mm/Hg), mean (SD)	139.0 (19.6)	139.5 (19.8)	136.2 (18.5)	0.005	133.4 (17.5)	137.3 (19.0)	135.0 (17.3)	0.310
Diastolic BP (mm/Hg), mean (SD)	84.4 (16.6)	84.6 (17.5)	83.2 (9.3)	0.165	81.6 (7.9)	83.5 (9.2)	83.4 (10.4)	0.381
Diabetes mellitus (%)	609 (26.7)	547 (28.0)	62 (18.8)	0.001	10 (17.9)	39 (18.4)	13 (21.0)	0.884
Insulin therapy (%)	114 (5.2)	106 (5.7)	8 (2.5)	0.016	1 (1.8)	6 (2.9)	1 (1.6)	0.804

Variables with missing values >5% (Smoking 5.3%, Alcohol 6.5%, Waist 6.5%); AGB = adjustable gastric banding; SG = sleeve gastrectomy; RYGB = Roux-en-Y gastric bypass; BP = blood pressure.

**Table 2:** Association (HR and 95% CI) between baseline characteristics, bariatric surgery (as time dependent variable) and all-cause mortality.

	Crude Effect		Adjusted Effect *	
	HR (95% CI)		HR (95% CI)	
	N = 2285	*p*	N = 2218 ^	*p*
Bariatric Surgery	0.36 (0.19–0.67)	<0.001	0.52 (0.27–0.98)	0.044
Adjustable gastric banding	NE	-	NE	-
Sleeve gastrectomy	0.28 (0.12–0.63)	<0.001	0.39 (0.17–0.89)	0.025
Roux-en-Y gastric bypass	0.62 (0.23–1.68)	0.347	0.97 (0.35–2.66)	0.959
Age (years)	1.07 (1.06–1.09)	<0.001	1.07 (1.05–1.08)	<0.001
Females	0.49 (0.38–0.64)	0.001	0.54 (0.41–0.70)	<0.001
Educational level				
Degree	1.00		1.00	
High school	0.73 (0.33–1.62)	0.446	0.92 (0.41–2.05)	0.835
Intermediate school	1.13 (0.53–2.43)	0.746	1.04 (0.48–2.26)	0.915
Elementary school or less	1.80 (0.83–3.91)	0.138	1.12 (0.51–2.47)	0.776
Smoking				
No	1.00		1.00	
Yes	1.27 (0.96–1.68)	0.101	1.60 (1.19–2.14)	0.002
not defined	1.13 (0.70–1.83)	0.608	1.05 (0.64–1.74)	0.843
Charlson Index				
0	1.00		1.00	
1	2.16 (1.62–2.88)	<0.001	1.38 (1.00–1.89)	0.048
≥2	4.58 (3.30–6.35)	<0.001	2.26 (1.51–3.39)	<0.001
BMI (kg/m^2^)	1.03 (1.02–1.03)	<0.001	1.02 (1.01–1.03)	<0.001
Systolic blood pressure				
≤140 mmHg	1.00		1.00	
>140 mmHg	1.61 (1.25–2.08)	0.234	1.14 (0.87–1.49)	0.329
Fasting glucose				
<126 mg/dL	1.00		1.00	
≥126 mg/dL	2.84 (2.18–3.70)	<0.001	1.34 (0.96–1.86)	0.084
Total cholesterol				
167–218 mg/dL **	1.00		1.00	
≤166 mg/dL	1.94 (1.44–2.62)	<0.001	2.02 (1.49–2.75)	<0.001
≥219 mg/dL	1.38 (1.01–1.89)	0.044	1.17 (0.84–1.62)	0.358
Triglycerides				
<150 mg/dL	1.00		1.00	
≥150 mg/dL	1.44 (1.12–1.86)	0.005	1.04 (0.87–1.49)	0.329

Test for interaction: age-BS *p* = 0.414; diabetes-BS *p* = 0.279; HR = hazard ratio; CI = confidence interval; NE= Not Estimable; * Covariates effects were estimated by the multivariable model including BS as a single category; ^ patients without missing values in covariates were included in the model, except for smoking, for which a separate category (not defined) was created, since 5.3% of smoking data were missing; ** cholesterol interquartile range.

**Table 3:** Characteristics and rate of hospitalizations by bariatric surgery.

	Non-BS Group	BS Group	
	Hospitalizations	Pre-BS Hospitalizations	Post-BS Hospitalizations
	(N = 8697)	(N = 1013)	(N = 711)
Person-years	18,973.6	1654.1	1459.6
Number of hospital admissions			
Total hospitalization rate per person/years	0.46	0.61	0.49
Hospitalization rate–medical per person/years	0.37	0.53	0.25
Hospitalization rate–surgical per person/years	0.09	0.08	0.23
Length of stay (days), median (IQR)	14 (6–27)	14 (4–28)	6 (3–13)
Weight of DRG, mean (SD)	0.96 (0.7)	0.76 (0.4)	1.13 (0.6)
Total DRG tariffs per person/years (€)	2075	2508	1891

SD = Standard Deviation; IQR = interquartile range; DRG = diagnosis-related group; hospitalization for BS excluded (N = 331).

**Table 4:** Risk of hospitalization by bariatric surgery (upper part) and by type of surgery (lower part).

	HR	95% CI	*p*
**Risk of hospitalization between non-BS group and BS group (post-surgery)**			
Non-BS patients	1.00		
BS patients			
before surgery	1.16	1.08–1.24	<0.001
after surgery	0.81	0.72–0.90	<0.001
**Risk of hospitalization among non-BS group and types of BS (pre-and post-surgery)**			
Non-BS group (reference category)	1.00		
Adjustable gastric banding			
before surgery	1.14	0.96–1.34	0.131
after surgery	0.92	0.72–1.18	0.499
Sleeve gastrectomy			
before surgery	1.13	1.04–1.22	0.004
after surgery	0.77	0.68–0.88	<0.001
Roux-en-Y gastric by-pass			
before surgery	1.30	1.15–1.47	<0.001
after surgery	0.78	0.63–0.98	0.032
**Risk of hospitalization by types of BS (pre- and post-surgery)**			
Adjustable gastric banding			
pre-surgery	1.00		
post-surgery	0.43	0.26–0.72	0.001
Sleeve gastrectomy			
pre-surgery	1.00		
post-surgery	0.57	0.46–0.71	<0.001
Roux-en-Y gastric by-pass			
pre-surgery	1.00		
post-surgery	0.51	0.34–0.77	0.001

## Data Availability

The dataset analyzed during the current study is available from the corresponding author on reasonable request.

## Supplementary Materials

The following are available online at https://www.mdpi.com/article/10.3390/nu13093150/s1, Figure S1: Cumulative incidence of bariatric surgery (with 95% banding curves) in the whole cohort (considering mortality as competitive risk), Table S1: Socio-demographic and clinical predictors (SHR and 95% CI) of bariatric surgery (considering mortality as competitive risk), Figure S2: Overall survival of the whole cohort (A) and by bariatric surgery (B) considered as time dependent variable.
