# Supplementary material for: Effect of Bariatric Surgery on Survival and Hospitalizations in Patients with Severe Obesity. A Retrospective Cohort Study
Source: Nutrients. 2021 Sep 9;13(9):3150. doi: 10.3390/nu13093150 (PMC8464847; doi:10.3390/nu13093150)
Supplement: Supplementary file 1 [file nutrients-13-03150-s001.zip › nutrients-1374955-supplementary.pdf]

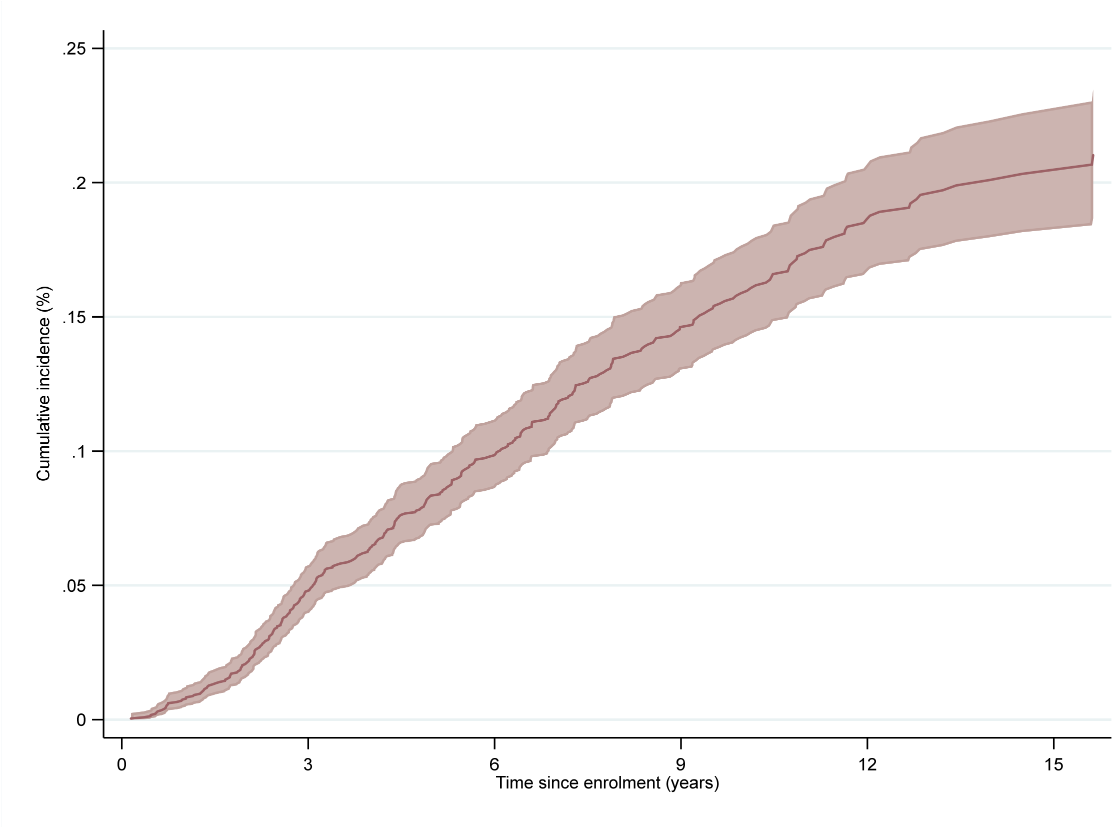

Figure S1. Cumulative incidence of bariatric surgery (with 95% banding curves) in the whole cohort (considering mortality as competitive risk).

Table S1. Socio-demographic and clinical predictors (SHR and 95% CI) of bariatric surgery (considering mortality as competitive risk).

	**Crude effect**<br>SHR (95% CI)*<br>N=2,285	p	**Adjusted effect**<br>SHR (95% CI)*<br>N = 2,218^	p
Age (years)	0.97 (0.96-0.98)	<0.001	0.97 (0.96-0.98)	<0.001
Females	1.15 (0.91-1.45)	0.252	1.17 (0.91-1.50)	0.211
Educational level				
Degree	1		1	
High school	0.96 (0.53-1.72)	0.884	0.95 (0.51-1.74)	0.856
Intermediate school	1.07 (0.60-1.89)	0.819	1.12 (0.62-2.03)	0.708
Elementary school or less	0.46 (0.24-0.90)	0.024	0.62 (0.31-1.25)	0.183
Smoking				
no	1		1	
yes	0.88 (0.68-1.13)	0.323	0.81 (0.63-1.06)	0.124
not defined	0.26 (0.12-0.58)	0.001	0.27 (0.12-0.61)	0.001
Charlson Index				
0	1		1	
1	0.67 (0.51-0.88)	0.845	0.81 (0.60-1.09)	0.165
≥2	0.71 (0.68-1.08)	0.107	0.96 (0.58-1.58)	0.866
BMI (kg/m^2)	1.01 (1.00-1.02)	0.073	1.01 (1.00-1.02)	0.016
Systolic blood pressure				
≤140 mmHg	1		1	
>140mmHg	0.77 (0.61-0.99)	0.039	0.89 (0.70-1.15)	0.356
Fasting glucose				
<126 mg/dL	1		1	
≥126mg/dL	0.67 (0.48-0.94)	0.021	0.93 (0.62-1.39)	0.384
Total cholesterol				
167-218 mg/dL**	1		1	
≤166 mg/dL)	0.97 (0.75-1.27)	0.845	0.92 (0.70-1.20)	0.545
≥219 mg/dL)	0.91 (0.69-1.19)	0.477	1.07 (0.80-1.43)	0.657
Triglycerides				
<150 mg/dL	1		1	
≥150 mg/dL	0.96 (0.77-1.20)	0.734	1.07 (0.83-1.38)	0.611

*SHR= sub-distribution hazards ratio; CI=confidence interval; ^patients without missing values in covariates were included in the model, except for smoking, for which a separate category (not defined) was created, since 5.3% of smoking data were missing; **Cholesterol Interquartile range

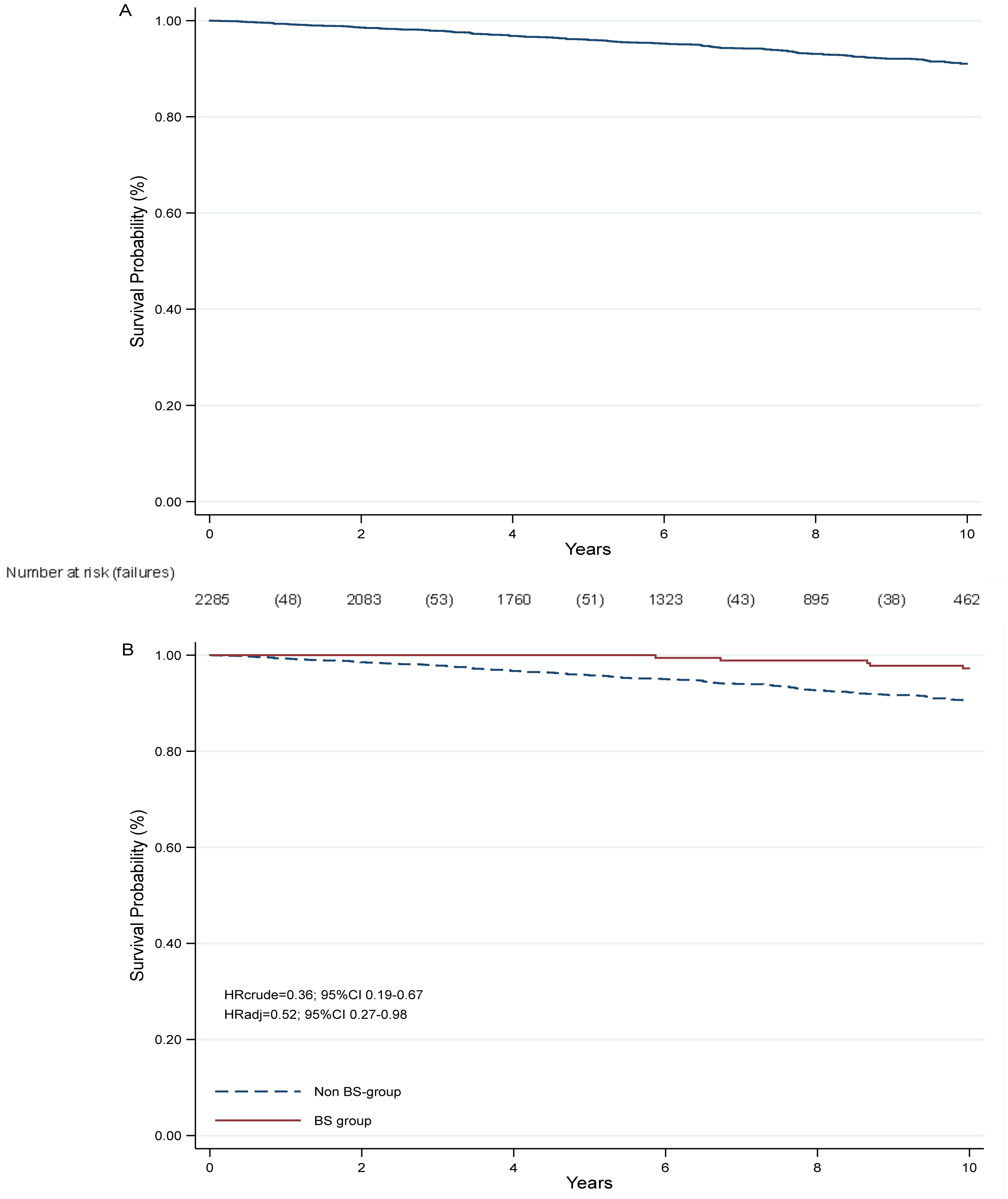
A
Survival Probability (%)
1.00
0.80
0.60
0.40
0.20
0.00
0
2
4
6
8
10
Years
Number at risk (failures)
2285 (48) 2083 (53) 1760 (51) 1323 (43) 895 (38) 462
B
Survival Probability (%)
HRcrude=0.36; 95%CI 0.19-0.67
HRadj=0.52; 95%CI 0.27-0.98
Non BS-group
BS group
Years

Figure S2. Overall survival of the whole cohort (A) and by bariatric surgery (B) considered as time dependent variable .
